# Physiological adaptations to serpentinization in the Samail Ophiolite, Oman

**DOI:** 10.1038/s41396-019-0391-2

**Published:** 2019-03-12

**Authors:** Elizabeth M. Fones, Daniel R. Colman, Emily A. Kraus, Daniel B. Nothaft, Saroj Poudel, Kaitlin R. Rempfert, John R. Spear, Alexis S. Templeton, Eric S. Boyd

**Affiliations:** 1 Department of Microbiology and Immunology, Montana State University, Bozeman, MT USA; 2 NASA Astrobiology Institute, Mountain View, CA USA; 3 Department of Civil and Environmental Engineering, Colorado School of Mines, Golden, CO USA; 4 Department of Geological Sciences, University of Colorado, Boulder, CO USA

**Keywords:** Water microbiology, Microbial ecology, Biogeochemistry

## Abstract

Hydration of ultramafic rock during the geologic process of serpentinization can generate reduced substrates that microorganisms may use to fuel their carbon and energy metabolisms. However, serpentinizing environments also place multiple constraints on microbial life by generating highly reduced hyperalkaline waters that are limited in dissolved inorganic carbon. To better understand how microbial life persists under these conditions, we performed geochemical measurements on waters from a serpentinizing environment and subjected planktonic microbial cells to metagenomic and physiological analyses. Metabolic potential inferred from metagenomes correlated with fluid type, and genes involved in anaerobic metabolisms were enriched in hyperalkaline waters. The abundance of planktonic cells and their rates of utilization of select single-carbon compounds were lower in hyperalkaline waters than alkaline waters. However, the ratios of substrate assimilation to dissimilation were higher in hyperalkaline waters than alkaline waters, which may represent adaptation to minimize energetic and physiologic stress imposed by highly reducing, carbon-limited conditions. Consistent with this hypothesis, estimated genome sizes and average oxidation states of carbon in inferred proteomes were lower in hyperalkaline waters than in alkaline waters. These data suggest that microorganisms inhabiting serpentinized waters exhibit a unique suite of physiological adaptations that allow for their persistence under these polyextremophilic conditions.

## Introduction

The earliest forms of life on Earth are commonly thought to have relied on redox reactions involving hydrogen (H_2_) and single-carbon (C1) compounds to fuel their carbon and energy metabolisms [1]. The geologic process of serpentinization may have served as a source of these substrates for early life [2]. During serpentinization, the oxidation of ferromagnesian minerals (e.g., olivine) in mafic and ultramafic rocks couples with the reduction of water to produce H_2_ [3]. High concentrations of dissolved H_2_ can drive the reduction of dissolved inorganic carbon (DIC) to reduced carbon compounds such as formate (HCOO^−^), carbon monoxide (CO), and methane (CH_4_) through reactions similar to the Sabatier process [3, 4]. However, increased serpentinization reaction progress also yields increasingly high pH waters that can be metal-rich and limited in available oxidants [5]. Moreover, divalent cations in serpentinized waters and in minerals common in ultramafic rocks interact with CO_2_ to precipitate mineral carbonates (e.g., MgCO_3_, CaCO_3_), thereby yielding low concentrations of DIC in highly reacted waters [5–7]. Despite the potential for serpentinization to have fueled the metabolisms of life on early Earth, the specific adaptations that allow for life under these conditions are not well understood.

Previous studies of serpentinizing systems have shown low densities of microbial cells (often <10^6^ cells mL^−1^) in environments impacted by serpentinization [8–12]. In serpentinite springs of the Voltri Massif, Italy, Cabeço de Vide, Portugal, and the Cedars peridotite body, California, USA, as few as 10^2^ cells mL^−1^ were detected [13–15]. Despite relatively low densities, microbial cells inhabiting environments influenced by serpentinization appear to be capable of using products of this process. For example, sequencing of community DNA extracted from a hyperalkaline seep community in the Tablelands Ophiolite (Newfoundland, Canada), a hydrothermal chimney community at Lost City Hydrothermal Field (LCHF; mid-Atlantic Ocean), and the Coast Range Ophiolite Microbial Observatory (CROMO; California, USA) revealed genes that code for a variety of hydrogenase and CO dehydrogenase (CODH) proteins [9, 16], suggesting that organisms within these communities are capable of utilizing H_2_ and CO in their carbon and/or energy metabolisms. Similarly, biological ^13^CO consumption was observed in microcosm experiments containing water and sediments from hyperalkaline springs in the Tablelands Ophiolite [17]. Additional metagenomic sequencing data coupled with experimental evidence demonstrating transformation of isotopically labeled acetate (^13^CH_3_COO^−^) to methane (^13^CH_4_), as well as ^13^CH_4_ transformation to isotopically labeled bicarbonate (H^13^CO_3_^−^), points to active CH_4_ cycling in serpentinite springs of the Voltri Massif, Italy [13]. A high relative abundance of 16S ribosomal RNA (rRNA) genes closely related to known methanogens (up to 90% of the total archaeal population) coupled with detection of all key genes involved in hydrogenotrophic methanogenesis, acetoclastic methanogenesis, and formatotrophic methanogenesis in metagenomes from the Santa Elena Ophiolite, Costa Rica, reveals the putative importance of methanogens in this and potentially other environments undergoing active serpentinization [8]. Similarly, physiological inference based on homology of 16S rRNA genes suggests that organisms capable of H_2_ oxidation, CO oxidation, and CH_4_ cycling inhabit a serpentinizing seep in the Zambales Ophiolite, Philippines [18], and putative H_2_ and CH_4_ oxidizers have similarly been identified via homology of 16S rRNA genes in a serpentinite-hosted seep at the Chimaera Ophiolite, Turkey [19]. The isotopic signature of lipid biomarkers combined with metagenomic insights suggest that HCOO^−^ serves as a source of electrons for sulfate reducers, but not methanogens, in the LCHF [20]. Together, these data suggest that microbial communities from globally distributed environments impacted by serpentinization harbor the functional capacity to utilize products of serpentinization to support their energy and carbon metabolisms.

The Samail Ophiolite in the Sultanate of Oman is the largest, best-exposed ophiolite in the world, thereby providing an accessible field location for studying the physiological adaptations that allow microorganisms to inhabit environments impacted by serpentinization [21]. The ophiolite is composed of both mafic and ultramafic rocks (largely gabbros and peridotites, respectively) that are purported to be undergoing active serpentinization, resulting in distinct water types influenced by host bedrock lithology, mixing, and the extent of water–rock interactions [5]. Rempfert et al. examined the influence of geochemistry on the taxonomic composition of microbial communities in fracture waters sampled from wells drilled in the Samail Ophiolite via 16S rRNA gene sequencing [5]. The results indicate that the pH of waters influenced microbial diversity and shaped the taxonomic composition of microbial communities, with communities inhabiting hyperalkaline (pH > 10) fracture waters exhibiting distinct taxonomic assemblages and lower diversity as compared with those from alkaline and circumneutral (pH ~7–10) waters. Moreover, physiological inferences based on homology of 16S rRNA gene sequences to cultivated representatives suggested that the metabolisms supporting dominant populations inhabiting hyperalkaline waters include the anaerobic, low energy-yielding processes of methanogenesis, acetogenesis, and fermentation [5, 22]. However, aside from these observations, it remains unclear how variation in the geochemical composition of waters in the Samail Ophiolite influences the abundances of microbial cells, their functional potential, and their rates of substrate utilization/production. This, in turn, limits understanding of the adaptations that enable microbial life to inhabit environments impacted by serpentinization.

In the present study, we examined microbial communities in fracture waters sampled from subsurface wells in the Samail Ophiolite that span a pH gradient of approximately 7.6–11.3. This pH gradient serves as a general proxy for serpentinization reaction progress [5, 23], with waters with circumneutral pH (~7.6–10) having more recently infiltrated the ophiolite, and hyperalkaline (pH > 10) waters representing a contrasting fluid type whose composition has likely been influenced by long-term interaction with minerals in the ophiolite [5]. We subjected waters to geochemical analyses and planktonic communities to cell enumeration, metagenomic sequencing, and determination of potential rates of CO, HCOO^−^, and HCO_3_^−^ dissimilation (oxidation or reduction) and assimilation to biomass. Insights into the abundance, potential activities, and inferred genomic features of microorganisms in serpentinized waters are discussed in the context of possible adaptations allowing life to persist in polyextremophilic conditions that may be reminiscent of those on early Earth.

## Materials and methods

### Site description, water sampling

The classification scheme used here to describe major water types in the Samail Ophiolite, Sultanate of Oman, has been reported previously [5]. A submersible pump was used to collect water samples in February 2017 from seven previously drilled wells in the Samail Ophiolite (Table 1). Briefly, waters were collected from beneath the air–water interface in each well at depths specified in Table 2, including two depths for NSHQ14: 50 m (NSHQ14B) and 85 m (NSHQ14C). After pumping ~100 liters of water through the tubing, biomass was collected for DNA extraction using in-line 0.2 µm Millipore polycarbonate filters in 47 mm Pall polycarbonate filter housings.

**Table 1:** Locations of the wells that were sampled in 2017 for this study and the bedrock type that hosts them

Well name	WAB188	WAB105	WAB104	WAB55	NSHQ4	WAB71	NSHQ14B/C
pH	7.6^a^	8.3^a^	8.5	9.2	10.5^b^	10.6	11.1/11.3
UTM Easting	671,123	644,678	643,099	634,777	670,971	670,322	675,495
UTM Northing	2,529,798	2,536,524	2,541,124	2,506,101	2,531,699	2,533,981	2,529,716
Bedrock type	Contact	Peridotite	Peridotite	Contact	Contact	Peridotite	Peridotite
Fluid type	Contact	Alkaline peridotite	Alkaline peridotite	Contact	Hyperalkaline peridotite	Hyperalkaline peridotite	Hyperalkaline peridotite
Elevation (m)	514	688	842	531	514	608	526

Bedrock type is as reported in Rempfert et al. [5]. A “contact” bedrock type describes wells that are near the contact between gabbro-dominated and peridotite-dominated lithologies. The pH of NSHQ14B (50 m depth) and NSHQ14C (85 m depth) are separated by a slash. The elevation of each well is listed in meters above sea level

^a^ Data collected from wells in 2016 are reported since measurements were not collected during the 2017 field season

^b^ Data collected from wells in 2015 are reported since measurements were not collected during the 2016 or 2017 field seasons

**Table 2:** Description of field measurements of well waters collected in 2017

Well	WAB188	WAB105	WAB104	WAB55	NSHQ4	WAB71	NSHQ14B (50 m)	NSHQ14C (85 m)
Fluid type	Contact	Alkaline	Alkaline	Contact	Hyperalkaline	Hyperalkaline	Hyperalkaline	Hyperalkaline
pH	7.6^a^	8.3^a^	8.5	9.2	10.5^b^	10.6	11.1	11.3
Water level (m)	9	–	35	8	5	8	12	12
Pump depth (m)	–	50	70	30	6	50	50	85
Temp. (°C)	33.0^a^	31.6^a^	33.4^a^	34.7^a^	33.3^b^	34.5^a^	34.4	36.3
Cond. (μs cm^−1^)	926^a^	448^a^	493^a^	1171^a^	1249^b^	1803^a^	493^a^	493^a^
ORP (mV)	214	178^a^	180^a^	110^a^	−342^b^	−86^a^	−415	−253

Water level and pump depth level refer to the number of meters below the top of the well casing that water was encountered and where water was pumped from, respectively. A (–) indicates that data were not collected for waters from the specified well. All “alkaline” and “hyperalkaline” wells are hosted by peridotite bedrock

^a^ Data collected from wells in 2016 are reported since measurements were not collected during the 2017 field season

^b^ Data collected from wells in 2015 are reported since measurements were not collected during the 2016 or 2017 field seasons

Quantification of major cations (SO_4_^2−^, NO_3_^−^) via ion chromatography and ferrous iron concentrations were collected as described previously [5]. Water temperature, conductivity, pH, oxidation-reduction potential (ORP), and the concentration of dissolved oxygen were measured in the field with a Hach (Loveland, CO) HQ40D Portable Multi Meter [5]. Water samples for dissolved gas analysis were collected via the bubble strip method described previously [24]. Methods for determining DIC concentrations and dissolved gas concentrations are reported in the Supplemental Online Materials.

### DNA extraction and shotgun metagenomic sequencing

Filtered biomass was subjected to DNA extraction using a Zymo (Irvine, CA) Research Xpedition Soil/Fecal DNA MiniPrep Extraction kit according to manufacturer instructions. Triplicate DNA extractions were pooled for metagenomic library preparation, quantified, and normalized to a total of 1 ng for library preparation using the Nextera XT library preparation kit (Illumina Inc., San Diego, CA). DNA from WAB71 and WAB105 were below the 1 ng threshold and were thus bead-cleaned with Kapa Pure Beads (Roche, Pleasanton, CA) to bind all fragmented DNA of 150 bp in length or more. DNA was then concentrated by elution off the beads into a smaller volume of nuclease-free water before library preparation using the Nextera protocols. Following tagmentation and amplification, products were pooled at equimolar concentrations and sequenced on the Illumina HiSeq 2500 Rapid Run platform (2 × 250 bp). Raw sequence reads were quality filtered, trimmed of adapters, and assembled as described in Supplemental Online Materials.

### Comparisons of genomic functional potential

To assess differences in functional potential among metagenomes, encoded proteins (>10 amino acids) inferred from metagenomes were clustered into putative homologous protein family bins (>30% amino-acid identity) using CD-HIT v.4.6 [25], following previously described methods [26]. Representative proteins from the 30% protein bin clusters were annotated against the Kyoto Encyclopedia of Genes and Genomes (KEGG) function database [27] using the KEGG Automatic Annotation Server [28]. A subset of the KEGG orthology (KO) assignments were extracted that encompassed the “Metabolism” functional subcategory (*n* = 10,628), and the abundances of these KOs were used to construct a table of protein bin counts within metagenomes using custom MATLAB scripts (scripts available at: https://github.com/dcolman1/matlab_scripts). The abundance table was then subjected to principal coordinate analysis in R [29] following normalization to total metagenome protein counts, as described previously [26].

### Enrichment of functional genes and correlational analyses among metagenomes

Correlational analyses were conducted to determine the association of annotated proteins involved in energy metabolism to overall differences in protein-coding gene profiles. Statistically significant (*p* < 0.05) correlations of “Energy Metabolism” KO abundances with protein-coding gene dissimilarities were determined using the “env.fit” function within the vegan R package [30]. The distribution of target protein-coding functional genes (formate dehydrogenases [FDH], CODHs, methyl coenzyme reductases [MCRs], and acetyl-CoA synthases [ACSs]) were assessed by querying the metagenomes using BLASTp with bait sequences for the active site subunits for each of the proteins or protein complexes. The specified proteins were chosen as targets to identify and characterize the distribution/abundance of populations putatively involved in the metabolism of HCOO^−^, CO, and HCO_3_^−^/CH_4_, as determined via microcosm-based activity assays (described below). Positive matches within the metagenome databases were considered as those with an *E*-value < 1 × 10^−6^, >30% amino-acid homology, and >60% of the length of the BLASTp bait sequence. The number of homologs detected in each metagenome was then normalized to total assembly size. Functional gene homologs were assigned taxonomic identities by BLAST querying the functional genes against the IMG database [31].

### Estimation of metagenome-assembled genome (MAG) sizes

Genome completeness and MAG sizes (in Mbp) were estimated using the CheckM software package (v.1.0.5) [32]. Estimated genome sizes were then extrapolated from the draft MAG sizes and percent estimated completion of sufficiently complete MAGs (>40% estimated completeness). Relative abundances were estimated for each population represented by the MAGs, based on the percentage of raw reads mapped to each MAG using the “profile” command within CheckM.

### Oxidation state of carbon in inferred proteomes

The average oxidation state of carbon (Z_c_) was calculated for each protein sequence encoded by the eight metagenomes assembled for this study based on an algorithm described previously [33] using a custom python script (script available at: https://github.com/spoudel1/Oxidation_state_of_carbon/blob/master/oxidationstate.py). CheckM was used to obtain the read coverage for each contig, and the average read coverage for each protein sequence was then determined by the contig read-mapping quantification described above. The Z_c_ of each protein sequence was then weighted by protein length and the average read coverage of the genes encoding the specified protein sequences using a custom python script (script available at: https://github.com/spoudel1/Oxidation_state_of_carbon/blob/master/readfile.py).

### Enumeration of planktonic cells

Five wells (WAB105, WAB104, WAB55, WAB71, and NSHQ14) were selected as targets for cell counts to include three representative water types spanning a pH gradient from ~8 to 11. Subsamples of homogenized waters were preserved in the field by addition of 0.22 µm filtered formaldehyde to a final concentration of 10% vol./vol.

Samples were maintained at ambient temperature during transport to the laboratory and were then placed at 4 °C for storage. Stored samples were homogenized and an aliquot of water was stained with 4′,6-diamidino-2-phenylindole (DAPI) and counterstained with SYBR Gold nucleic acid stains (Invitrogen, Carlsbad, CA). Stained cells were filtered onto 0.22 μm Isopore black polycarbonate membrane filters (EMD Millipore, Burlington, MA) and viewed at 1000× under oil immersion for direct enumeration using an EVOS FL Imaging System fluorescent microscope (Thermo Fisher Scientific, Waltham, MA).

### Substrate transformation rate potentials

Waters from the same five wells as those chosen for cell counts were used to determine potential rates of transformation of select 1-carbon substrates via microcosm assays as described previously [34]. Ten milliliters of unfiltered water collected in pre-evacuated Cali-5 Bond bags (Calibrated Instruments, McHenry, MD) were injected into N_2_ purged, autoclaved, butyl rubber stoppered 24 mL serum bottles. Vials were prepared in triplicate and kept at ambient temperature during transport to the lab. Abiological controls were prepared in triplicate for each well as described above, except serum vials were inoculated with water that had been filtered (0.22 μm). Each microcosm vial contained a 1 mM final concentration of HCOO^−^, HCO_3_^−^, and CO with 5 µCi ^14^C-HCOO^−^, ^14^C-HCO_3_^−^, and ^14^C-CO. Following incubation at 37 °C, samples were acidified and headspace ^14^C-CO_2_ and ^14^C-biomass were measured as described previously [34]. ^14^C-CH_4_ measurements were performed by trapping a known volume of headspace gas using a specially fabricated scintillation vial containing a butyl rubber septum with Cytoscint ES scintillation cocktail. The radioactivity measured in counts per minute associated with each of the samples was measured on a PerkinElmer Tri Carb 2900TR Liquid Scintillation Analyzer (PerkinElmer, Waltham, MA), converted to disintegrations per minute using a quench curve, and used to calculate the maximum rates of biological substrate transformation.

## Results

### Site characterization and geochemistry

Subsurface waters were sampled from seven previously drilled wells intersecting gabbro or peridotite bedrock of the Samail Ophiolite in February 2017 (Table 1). The pH of waters ranged from 7.6 to 11.3 (Table 1). High pH waters exhibited negative ORP (Table 2) and were generally enriched in compounds that microorganisms could potentially use as reductants, such as H_2_ and CH_4_ (Table 3). However, high pH waters were generally depleted in compounds that microorganisms could potentially use as oxidants, such as SO_4_^2−^, NO_3_^−^, and DIC (Table 3).

**Table 3:** Description of geochemical measurements conducted on well waters sampled in 2017

Well	WAB188	WAB105	WAB104	WAB55	NSHQ4	WAB71	NSHQ14B (50 m)	NSHQ14C (85 m)	DL (μM)
pH	7.6^a^	8.3^a^	8.5	9.2	10.5^b^	10.6	11.1	11.3	–
Fe^2+^ (μM)	0.4	5.0	2.0	2.5	0.8	0.2	0.1	2.0	0.006
SO_4_^2−^ (μM)	1130	292	477	875	683	42	131	2	1.04
NO_2_^−^ (μM)	6	DL	DL	8	DL	14	17	16	2.17
NO_3_^−^ (μM)	118	135	123	143	3.0	2.5	DL	DL	1.61
H_2_ (μM)	0.92	DL	DL	DL	DL	0.51	21	164	0.45
CH_4_ (μM)	1.69	0.02	0.02	0.10	155	12.6	34.6	12.6	0.015
DIC (mM)	3.0	3.5	3.5	2.9	0.04	0.12	0.05	0.13	0.098

Potential sources of measured oxidants, reductants, and single-carbon compounds in well waters are reported. Detection limits (DL) are indicated in the far right column

*DL* detection limit in μM

^a^ Data collected from wells in 2016 are reported since measurements were not collected during the 2017 field season

^b^ Data collected from wells in 2015 are reported since measurements were not collected during the 2016 or 2017 field seasons

### Planktonic cell abundances

**Fig. 1:**
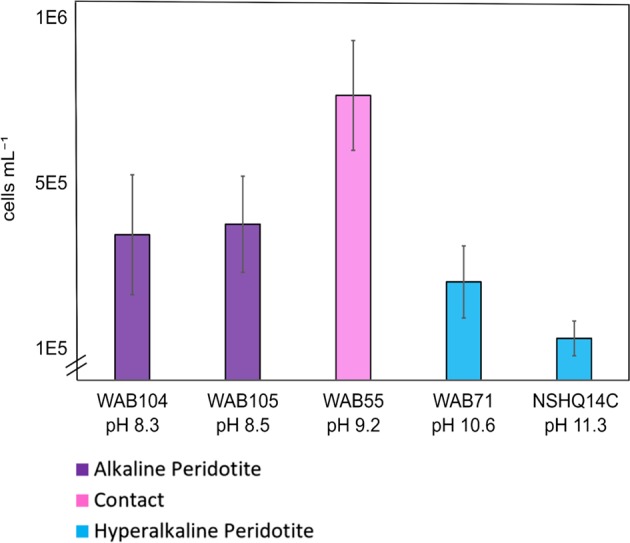
Planktonic cell concentration (cells mL^−1^) in subsurface waters sampled from wells in the Samail Ophiolite. The planktonic cell concentration in water sampled from each subsurface well is represented by a bar whose color corresponds to the legend at the bottom of the figure describing well water type. Subsurface well waters were filtered onto 0.22 µm membranes and cells were enumerated via epifluorescence microscopy (Table S3). Error bars reflect the standard deviation of three replicate subsamples with a minimum of 10 fields of enumeration

Planktonic cell concentrations were on the order of 10^5^ cells mL^−1^ in waters from wells sampled in 2017 (Fig. 1). Average cell concentrations were higher in waters sampled from alkaline peridotite wells (3.77 × 10^5^ cells mL^−1^ and 4.03 × 10^5^ cells mL^−1^ in WAB105 and WAB104, respectively) than in those sampled from hyperalkaline peridotite wells (2.58 × 10^5^ cells mL^−1^ and 1.16 × 10^5^ cells mL^−1^ in WAB71 and NSHQ14, respectively) (Table S3). The highest concentration of cells (7.28 × 10^5^ cells mL^−1^) was observed in the contact well, WAB55.

### Functional potential of microbial communities

**Fig. 2:**
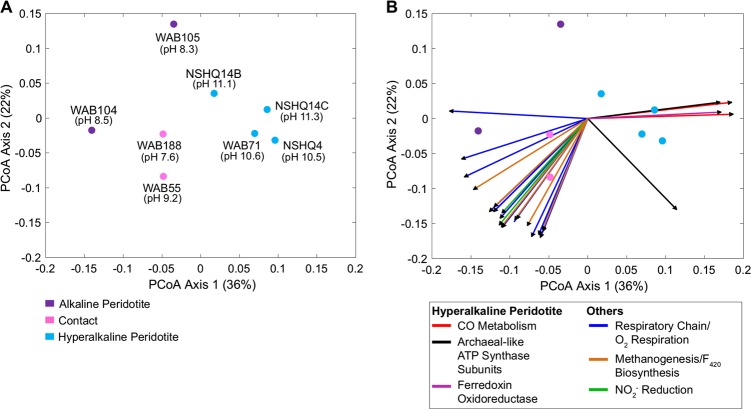
Similarity in protein-coding genes among eight metagenomes generated from communities sampled from well waters within the Samail ophiolite (**a**) correlated with protein-coding gene functions involved in energy metabolism (**b**). Each metagenome is represented by a filled circle that is colored by water type, as displayed in the legend below the plot. The percent variation explained by each axis is given in parentheses in each axis label (as indicated by relative eigenvalues). Ordination of the metagenomes is based on dissimilarity in protein-coding gene homolog families that were annotated using the KEGG database as being involved in “Metabolism.” Panel **b** displays the same ordination as shown in (**a**), overlaid with vectors representing KEGG orthology groups that were significantly (*p* < 0.05) associated with the overall differences in the functional composition of metagenomes. The vectors are colored according to broad metabolism categories displayed in the legend below the plot

Sixteen metagenome assemblies were produced from DNA extracted from filtered biomass collected from eight subsurface wells in 2015 (Table S1), and from seven subsurface wells including two different depths in well NSHQ14 in 2017 (Table S2). Ordination of matrices describing the dissimilarity in the composition and relative abundance of protein bins inferred from metagenomes revealed patterns of clustering that were consistent with water types and their associations with different host-rock lithologies (Fig. 2a, Fig. S1). For example, communities inhabiting hyperalkaline and contact waters formed clusters, indicating similar functional potential among communities from these water types. Furthermore, metagenomes from the two depths sampled from NSHQ14 (NSHQ14C: 85 m and NSHQ14B: 50 m) formed a cluster with metagenomes from other hyperalkaline waters but were distinct from each other (Fig. 2a). Finally, the communities from the wells containing alkaline peridotite waters (WAB105 and WAB104) exhibited highly dissimilar protein-coding potentials, despite similar geochemistry of the well waters (Fig. 2a).

### Enrichment of functional genes involved in C1 metabolism

The abundances of specific proteins were correlated with overall inferred proteome dissimilarity to identify functions that distinguished protein encoding potential between communities. The metagenomes from hyperalkaline peridotite wells exhibited enrichment of protein encoding genes that were distinct from those of the other water types (Fig. 2b). Of note was the enrichment of anaerobic, nickel-dependent CODH subunits (CdhA, CdhB) and other encoded proteins typically involved in anaerobic microbial metabolisms (archaeal-type ATP synthase subunits and the anaerobic sulfite reductase subunit A, AsrA) in the hyperalkaline peridotite hosted water communities. In contrast, genes encoding proteins that were enriched in the nonhyperalkaline peridotite well communities included those involved in respiratory and electron transport chains (NADH dehydrogenases Nuo and Ndh; succinate dehydrogenase Sdh; quinolcytochrome oxidoreductase Qcr; and Pet: required for proper assembly of the cytochrome oxidase), O_2_ respiration (cyclooxygenase: Cox; and cytochrome oxidase subunit I: Cyo), and dissimilatory NO_2_^−^ reduction (nitrite reductase: Nir). In addition, several genes coding for proteins involved in methanogenesis or F_420_ biosynthesis (a cofactor involved in methanogenesis) were enriched in the alkaline peridotite well communities.

Enrichment of certain functionalities suggested that the metabolism of C1 compounds potentially distinguished well communities (i.e., via CO utilization and methanogenesis).

**Fig. 3:**
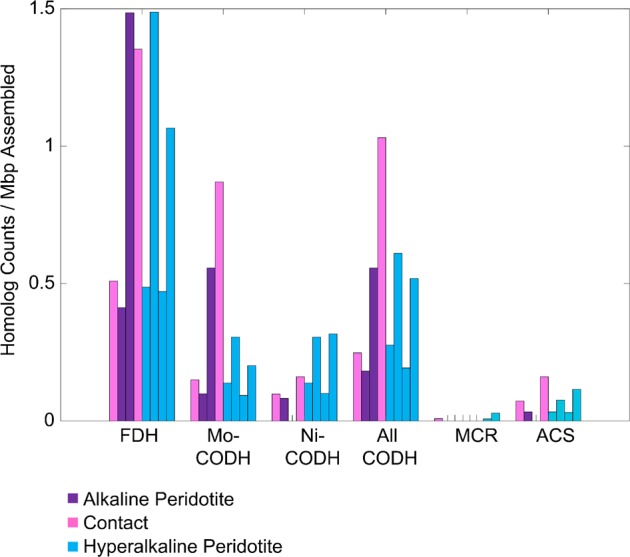
Enrichment of select functional genes associated with target one- or two-carbon metabolisms in metagenomes. Each bar represents one metagenome, ordered from left to right by increasing pH of the well waters that hosted these communities: WAB188, WAB105, WAB104, WAB55, NSHQ04, WAB71, NSHQ14B (50 m depth), NSHQ14C (85 m depth). Functional genes that were not detected in individual well metagenomes are indicated by tick marks where bars would otherwise be present. FDH, formate dehydrogenase; Mo-CODH, molybdenum-containing carbon monoxide dehydrogenase; Ni-CODH, Ni-containing CODH; MCR, methyl coenzyme M reductase; ACS, acetyl coenzyme A synthase

Consequently, a targeted assessment of the distribution and enrichment of genes coding for proteins involved in C1 metabolism was conducted. In general, FDH homologs were the most prevalent among the functional genes surveyed, followed by CODH, ACS, and MCR (Fig. 3). MCR homologs were detected in low abundance in the dataset and were identified in only three of the communities: those hosted by wells WAB188 and NSHQ14 (both samples B and C).

### Potential rates of C1 compound transformation

**Fig. 4:**
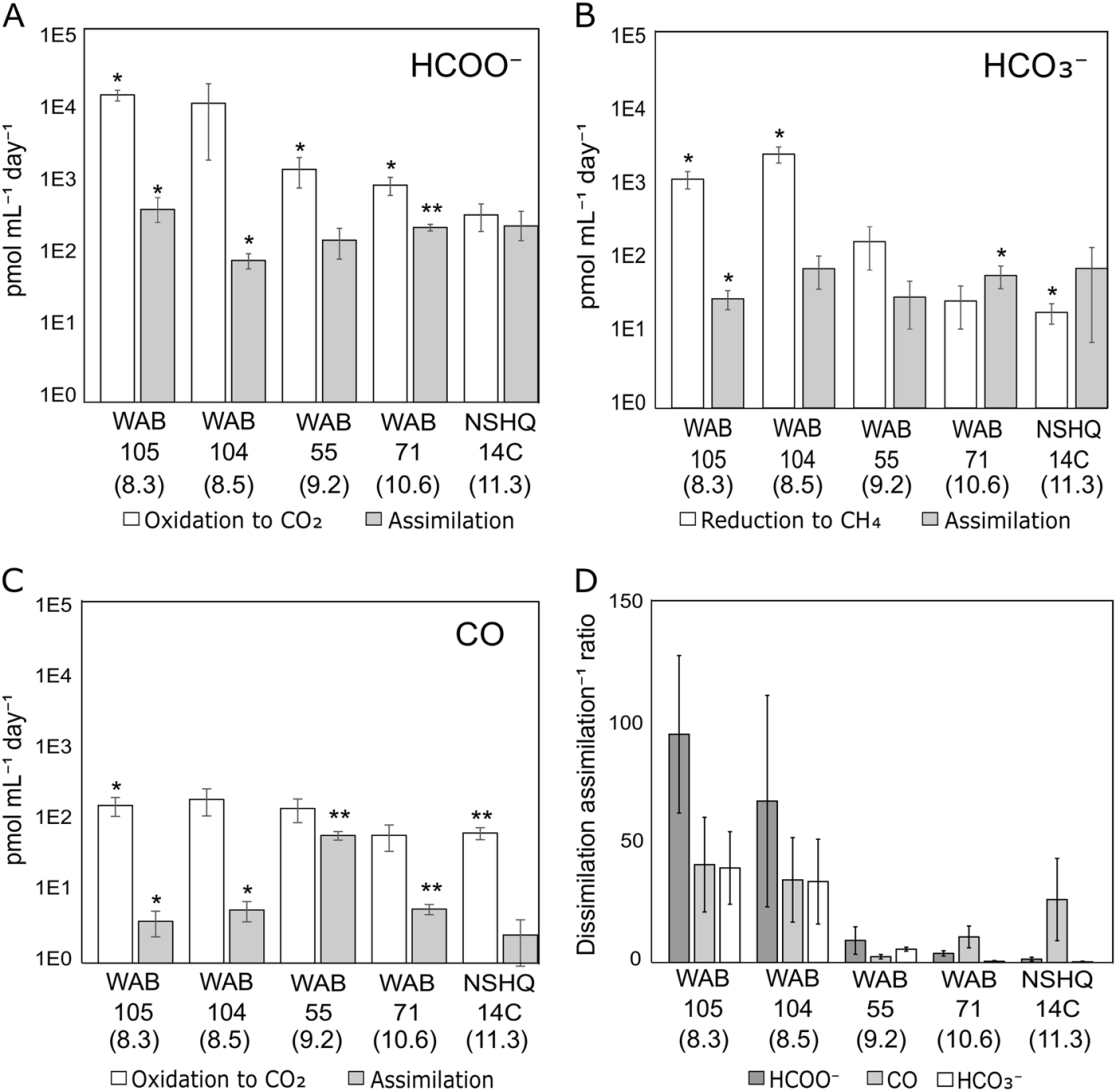
Maximum potential rates of biological assimilation and dissimilation (oxidation or reduction) of select one-carbon compounds by planktonic microbial communities in subsurface well waters collected from the Samail Ophiolite. Maximum potential rates of biological formate (HCOO^−^) oxidation to carbon dioxide (CO_2_) and assimilation of HCOO^−^ to biomass (**a**), bicarbonate (HCO_3_^−^) reduction to methane (CH_4_) and HCO_3_^−^ assimilation to biomass (**b**), carbon monoxide (CO) oxidation to CO_2_ and CO assimilation to biomass (**c**), and the ratio of the rate of substrate dissimilation (reduction or oxidation) to assimilation (to biomass) (**d**). The pH of each well is displayed in parentheses below the well name. Rates of substrate transformation were determined via microcosm assays using radiolabeled carbon tracers. Rates of substrate transformation in abiological controls were subtracted from biological assays to determine rates that are attributable to biology. Statistical significance of differences between biological assays and abiological controls were assessed via Student’s *t-*test assuming unequal variance for each condition (*p < 0.05, **p < 0.01). Error bars reflect the standard deviation of measurements of three replicate biological assays and three replicate abiological assays for each condition

Maximum potential rates of HCOO^−^, CO, and HCO_3_^−^ substrate transformation were determined via microcosm assays using ^14^C enriched substrates (Table S4). Significantly (*p* < 0.05) higher rates of substrate utilization were observed in biological assays as compared with abiological (0.22 µm filtered) controls in microcosms from multiple wells for all substrates and all transformations tested (Fig. 4). Rates of substrate use for energy generation (i.e., dissimilatory oxidation or reduction) were negatively correlated with pH for all substrates tested; but these differences were not statistically significant (*p* > 0.05) for reduction of HCO_3_^−^ to CH_4_ and for oxidation of HCOO^−^ to CO_2_ (Figs. 4a, b). However, rates of CO oxidation to CO_2_ exhibited a significant inverse linear correlation with the pH of well waters (Pearson *R* = −0.94, *p* < 0.05) (Fig. 4c).

Rates of substrate assimilation to biomass were also determined in the same microcosm assays. The rate of HCOO^−^ assimilation to biomass was correlated positively with pH (Pearson *R* = 0.90, *p* < 0.05); rates of assimilation of the other substrates were variable with respect to the pH of the water where the communities were sampled. The rate of CO assimilation to biomass was the highest in microcosms containing water from the contact well (WAB55) and was lower in microcosms containing waters from the alkaline and hyperalkaline peridotite wells. Rates of CO assimilation to biomass were the only metabolic rates that correlated positively with the concentration of cells in well waters (Pearson *R* = 0.88, *p* < 0.05). Rates of HCO_3_^−^ assimilation did not significantly vary with pH.

The ratios of rates of HCO_3_^−^ reduction to CH_4_ vs. rates of HCO_3_^−^ assimilation to biomass decreased in communities inhabiting increasingly high pH well waters (Pearson *R* = 0.87) (Fig. 4d). Similarly, the ratios of rates of HCOO^−^ oxidation to CO_2_ vs. rates of HCOO^−^ assimilation to biomass decreased among communities inhabiting increasingly high pH well waters (Pearson *R* = 0.84). No relationship was observed between ratios of the rate of CO assimilation to the rate of CO oxidation and well water pH.

### Estimated genome size and weighted oxidation state of carbon (Z_c_) in proteomes

**Fig. 5:**
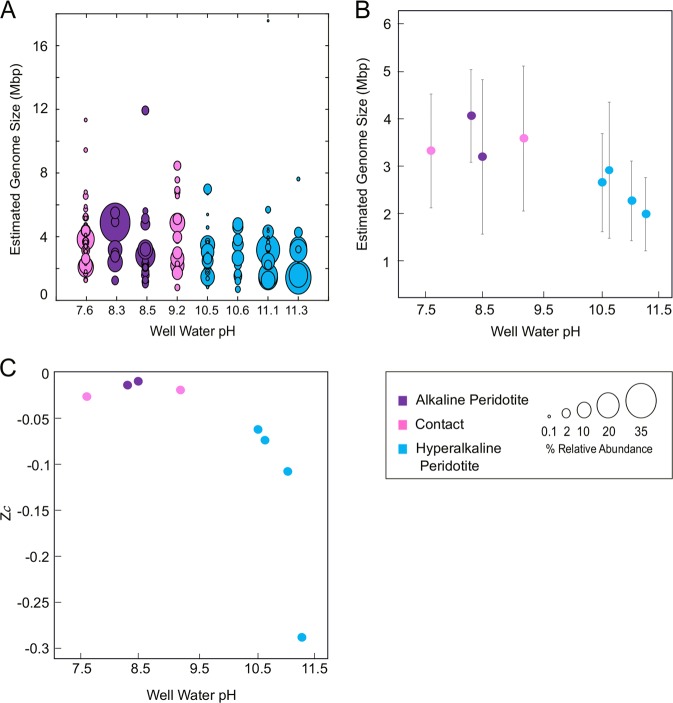
Features of metagenomes reflecting possible adaptations to hyperalkaline conditions. Plot depicting the distribution of estimated genome sizes (in Mbp) of metagenome-assembled genomes (MAGs) (**a**). MAGs are represented by circles ordered from left to right by increasing pH of the waters that hosted these communities. The size of each circle corresponds to the estimated relative abundance of each MAG, as indicated in the legend on the bottom right of the figure. Plot of median estimated genome sizes (in Mbp) of MAGs as a function of pH (**b**). Each point depicts the median estimated MAG size (in Mbp) for each well. Error bars depict the standard deviation of the estimated sizes of all MAGs with at least 40% estimated completeness within each metagenome. Plot of the weighted average oxidation state of carbon (Z_c_) in inferred proteomes of eight metagenomes as a function of pH (**c**). Each point represents the weighted Z_c_ value calculated for each metagenome based upon the Z_c_ per amino acid in the inferred protein sequences encoded by each metagenome. Z_c_ values were then normalized to protein length and read coverage of the gene encoding each specified protein and finally to the number of base pairs sequenced per metagenome

The mean estimated size of MAGs ranged from 4.0 Mbp (WAB105) to 2.1 Mbp (NSHQ14C) and exhibited a significant (Pearson *R* = −0.82, *p* < 0.05) inverse linear correlation with pH (Fig. 5b). Smaller MAGs from hyperalkaline well waters generally exhibited a larger relative abundance than those from alkaline well waters (Fig. 5a). The Z_c_ in inferred proteomes of MAGs ranged from −0.01 (WAB104) to −0.28 (NSHQ14C) and exhibited a significant (Pearson *R* = −0.72, *p* < 0.05) inverse linear correlation with the pH of the water from which the communities were sampled (Fig. 5c).

## Discussion

Wells intersecting gabbro and peridotite bedrock in the Samail Ophiolite provided access to subsurface waters with a range of geochemical compositions that can be interpreted to reflect the extent of serpentinization reaction progress and the degree of fluid mixing in each well [5]. These wells were drilled upwards of 20 years ago by the Oman Ministry of Regional Municipalities and Water Resources and are unlikely to still be experiencing the effects of drilling-related disturbances. The range of geochemical characteristics associated with these waters and the planktonic microbial communities they host thus provided an opportunity to examine the physiological adaptations that permit life in serpentinizing systems at the level of cell abundance, metabolic potential, potential rates of substrate transformation, and genomic characteristics.

Higher cell concentrations were observed in alkaline peridotite subsurface well waters than in hyperalkaline peridotite well waters (Fig. 1). This finding is consistent with previous reports of low cell concentrations in hyperalkaline waters of the Cedars peridotite body [13, 14] and in high H_2_ chimneys of the LCHF [11] but contrary to findings of higher cell concentrations in hyperalkaline waters compared with freshwaters associated with the Tablelands Ophiolite [10]. Geochemical analyses performed on hyperalkaline waters collected from the Samail Ophiolite revealed low concentrations of substrates that could potentially serve as oxidants for cells, including DIC (Table 3). Thus, relatively low cell numbers in hyperalkaline waters may be due, in part, to stress imposed on cells by high pH, as well as oxidant and inorganic carbon limitation in highly reacted waters, as has been suggested previously [35–37].

The highest concentration of cells was observed in the “contact” well (WAB55) (Fig. 1) that is hosted by peridotite but is within 1 km of the surface boundary between peridotite and gabbro (the paleo-crust-mantle transition) in the ophiolite [5]. The relatively high permeability of gabbro coupled with faulting observed at this boundary has been hypothesized to facilitate mixing between more oxidized gabbro-reacted waters and more reduced peridotite-reacted waters in the “contact” wells [5]. Mixing may provide microorganisms with higher concentrations of oxidants and reductants that are in disequilibrium when compared with alkaline or hyperalkaline waters, thereby providing additional chemical energy for cells to fuel their metabolisms [38] and thus to replicate. This in turn may promote a higher cell concentrations, a higher taxonomic diversity, as has been suggested previously [5], and a more robust and functionally diverse community like those recently suggested for hydrothermal environments sourced by mixing of end-member waters [39].

Distinct functional potentials inferred from metagenomes were observed among microbial communities sampled from well waters spanning a pH gradient from ~7.6 to 11.3 (Fig. 2a). This is consistent with clustering of microbial community compositions by water type, as noted previously based on 16S rRNA gene data [5]. Genes involved in anaerobic metabolisms were enriched in metagenomes from hyperalkaline waters (Fig. 2b), which is expected given the low concentrations of dissolved oxygen previously reported in these waters [5]. This observation is also consistent with previous physiological inferences from 16S rRNA genes extracted from waters in the Samail Ophiolite, which suggested that the metabolisms supporting dominant populations inhabiting hyperalkaline waters include the anaerobic processes of methanogenesis, acetogenesis, and fermentation [5]. Specifically, communities inhabiting hyperalkaline waters showed enrichment of genes encoding Ni-CODH in their metagenomes, enzymes that are typically associated with anaerobic Bacteria and Archaea [40, 41] including those that catalyze the processes of methanogenesis and acetogenesis [42]. In addition, other genes typically involved in anaerobic metabolisms were enriched in hyperalkaline waters, including the anaerobic sulfite reductase subunit A, AsrA, and archaeal-type ATP synthase subunits, the latter of which were also found to be enriched in a serpentinizing spring in The Cedars (California, USA) [37]. In contrast, community genomes from alkaline waters were enriched in genes coding for Mo-CODH, which is typically associated with aerobes [40, 41]. This finding is potentially consistent with the higher redox potential and the generally higher concentrations of dissolved oxygen in these waters [5].

Genes encoding FDH were highly abundant in all metagenomes and were more abundant than genes that encode proteins required to oxidize CO (i.e., Mo- or Ni-CODH) or required for the dissimilatory reduction of inorganic carbon via the processes of methanogenesis or acetogenesis (i.e., MCR, ACS; Fig. 3). Consistent with enrichment in FDH encoding genes, rates of HCOO^−^ assimilation or oxidation were higher than rates of assimilation or dissimilation of HCO_3_^−^ and CO from all well waters sampled (Fig. 4), suggesting that HCOO^−^ may be a preferred carbon and/or electron source among microorganisms in the Samail Ophiolite. The rates of HCOO^−^ assimilation to biomass were the only rates that correlated positively with the pH of waters where the communities were sampled (Fig. 4a). This indicates that microorganisms may be adapted to (i) efficiently utilize HCOO^−^ as a carbon source in DIC-limited hyperalkaline waters or (ii) take advantage of formate as a carbon/energy source in waters that may favor abiogenic formate production. The most abundant MAG (31% relative abundance) in the highest pH well, NSHQ14C, was inferred to encode an FDH protein that was most closely related to that of *Desulfitibacter alkalitolerans* (61% identity; Table S5), an anaerobic bacterium that can utilize HCOO^−^ as an electron donor and carbon source when paired with inorganic electron acceptors such as sulfite [43]. Abiotic CO_2_ reduction to HCOO^−^ is favored during the serpentinization of olivine under high H_2_ conditions [44]. Indeed, experimental low-temperature serpentinization of dunite from the Samail Ophiolite resulted in formation of HCOO^−^ and depletion of CO_2_ [45]. Thus, HCOO^−^ may serve as a bioavailable form of carbon under these conditions. Consistent with the observations presented here, prior work has indicated that abiogenic HCOO^−^ may serve as an important source of carbon for life in the DIC-limited environments associated with the LCHF, an alkaline serpentinizing system [20].

Despite previous evidence indicating that the process of methanogenesis is common in environments undergoing active serpentinization [12, 13, 18, 46–48], genes encoding subunits of MCR, an enzyme complex that catalyzes the final step of methanogenesis [49], were not highly abundant in metagenomes from waters sampled from the Samail Ophiolite (Fig. 3). The only metagenomes from alkaline or hyperalkaline environments where MCR was detected were from NSHQ14B and NSHQ14C. NSHQ14C also demonstrated significant rates of methanogenesis in microcosm assays (Fig. 4b). The inferred McrA protein homologs identified in the NSHQ14B and NSHQ14C metagenomes were related (88% identities) to those encoded by *Methanobacterium* sp. (Table S5), a hydrogenotrophic genus of methanogens [50]. Sequences closely related to *Methanobacterium* sp. were also reported in a 16S rRNA gene survey conducted on filtered waters from NSHQ14 in 2016 but were not detected in communities sampled from the same waters in 2014 and 2015 [5]. This may indicate temporally dynamic conditions in the well that fluctuate between those favoring or disfavoring growth of this strain. Genes encoding McrA were also detected in the metagenome from WAB188 (Fig. 3) and were 93% identical to those from *Methanobacterium* sp. (Table S5). 16S rRNA gene sequences affiliated with *Methanobacterium* sp. have been previously detected in filtered waters from this well sampled in 2015 but not in 2016 [5]. Significant rates of methanogenesis were observed in microcosms from wells WAB105 and WAB104 (Fig. 4b), where *mcrA* was not detected (Fig. 3). 16S rRNA genes affiliated with *Methanobacterium* were previously found to be widely distributed in well waters sampled from the Samail Ophiolite (16 of 20 samples) but were only abundant (>3%) in three samples. Thus, the lack of *mcrA* in metagenomes from WAB105 and WAB104 presented here may be due to undersampling of rare taxa.

The metagenome from the community inhabiting waters from WAB55 was enriched in genes coding for CODH, in particular those that coded for the “aerobic” Mo-CODH (Fig. 3). The most abundant MAG in WAB55 that was inferred to encode a Mo-CODH protein was most closely related to that encoded by the uncultured *Candidatus* Rokubacteria sp. (89% identity), which has been suggested to be a versatile mixotroph capable of growth under oxic or anoxic conditions [51]. This community also exhibited the highest rate of CO assimilation to biomass, but not CO oxidation (Fig. 4c). Previous results suggest that CO primarily served as a source of electrons rather than biomass carbon for organisms residing in a hyperalkaline environment influenced by serpentinization [17]. Consistent with this interpretation, rates of CO oxidation exceeded rates of CO assimilation in all microcosms containing waters from the Samail Ophiolite (Fig. 4c). However, CO assimilation was the only rate measured via microcosm assays that correlated significantly with observed cell concentrations in well waters (*p* < 0.05), suggesting that CO assimilation could be a major driver of biomass production in situ, especially in the mid-pH contact well, WAB55 (pH 9.2).

We hypothesized that carbon limitation in hyperalkaline wells would lead cells to assimilate a greater fraction of metabolized carbon than cells inhabiting circumneutral waters. To begin to test this hypothesis, we calculated the ratio of rates of HCOO^−^, HCO_3_^−^, and CO dissimilation (reduction or oxidation) to assimilation (to biomass) in microcosm assays. A generally negative relationship between the ratio of HCO_3_^−^ and HCOO^−^ dissimilation to assimilation and pH was observed, with the lowest ratios observed in communities in the contact and hyperalkaline well waters (Fig. 4d). This suggests that carbon limitation (substrate assimilation) may outweigh energy limitation (substrate dissimilation) for autotrophic populations in higher pH serpentinized waters in Oman. Importantly, high abundance members of hyperalkaline well communities were previously shown to be affiliated with putatively acetogenic organisms belonging to the candidate phylum OP1 that are likely to utilize the Wood–Ljungdahl (WL) pathway for carbon assimilation [5]. The WL pathway allows for assimilation of CO, HCOO^−^, or CO_2_ into biomass, so long as reductant is supplied by another component of cellular metabolism such as through H_2_ oxidation [52]. Homologs of genes encoding the ACS protein were detected in all metagenomes from hyperalkaline waters (Fig. 3). However, since we did not track production of ^14^C-acetate in the microcosm assays, it is possible that the low ratio of dissimilation to assimilation observed for HCO_3_^−^ and HCOO^−^ transformation is due to production of acetate. In addition, we did not track production of ^14^C-CH_4_ from ^14^C-HCOO^−^ or ^14^C-CO, however, *Methanobacterium* spp. can use both HCOO^−^ and CO as methanogenic substrates [50], and organisms capable of CH_4_ production from HCOO^−^ have previously been shown to inhabit a serpentinizing spring in the Cedars peridotite body, California and the Santa Elena Ophiolite, Costa Rica [8, 47]. Thus, it is also possible that the low ratio of dissimilation to assimilation observed for HCOO^−^ transformation is due to production of CH_4_. Additional work is needed to determine the primary processes supporting the energy and carbon metabolism of autotrophs inhabiting hyperalkaline well waters.

A significant inverse relationship was noted between the mean estimated size of MAGs reconstructed from communities inhabiting well waters in the Samail Ophiolite and the pH of those waters (Fig. 5b). This observation is consistent with genomic streamlining as a potential adaptation to energetic stress imposed on cells by the reducing, alkaline, inorganic carbon-limited conditions imposed by increased serpentinization reaction progress. In other nutrient limited or otherwise stressful environments, organisms have been suggested to encounter selection pressure to minimize the energetic costs associated with genome replication, costs that would be lessened by a reduction in genome size [53]. Indeed, members of a microbial community from a hyperalkaline spring in the Cedars were previously shown to comprise organisms with the smallest genomes reported for their respective taxa based on metagenomic inference [37]. Similarly, on the other end of the pH spectrum, the genomes of obligate acidophiles, which have been suggested to face chronic energy limitation [54], are relatively small compared with their neutrophilic counterparts [55]. This suggests that extremes in pH and potentially also nutrient limitation impose energetic stress on microbial populations, potentially leading to adaptation at the level of genome streamlining.

The average oxidation state of carbon in proteomes inferred from community metagenomes exhibited a significant negative correlation with increased pH of the well waters, with the lowest Z_c_ observed in NSHQ14C (Fig. 5c). A previous study conducted along an outflow channel of a hot spring also noted an inverse correlation between the Z_c_ in proteomes inferred from metagenomic data and the oxidation state of the local environment [33]. Similarly, the Z_c_ in proteomes inferred from the genomes of aerobic/facultatively anaerobic taxa (restricted to those involved in N_2_ fixation) were significantly higher than those from anaerobic taxa involved in N_2_ fixation, an observation that was attributed to the latter inhabiting environments with lower reduction potentials [56]. At a mechanistic level, the relationship between the Z_c_ of inferred proteomes and oxidation state of the local environment has been suggested to reflect selection to minimize energetic costs during protein synthesis [33]. In such a scenario, organisms inhabiting highly reduced environments, such as NSHQ14B and NSHQ14C, would be under selection to synthesize proteins that are comprised of amino acids that themselves are comprised of more reduced carbon, which, in turn, would minimize the overall energetic cost of protein synthesis. Indeed, MAGs recovered from NSHQ14B and NSHQ14C here and 16S rRNA genes recovered in a previous analysis of these waters [5] revealed communities dominated by putative acetogens and methanogens, organisms that are adapted to thrive in highly reduced environments [57].

In summary, the observations presented here suggest physiological adaptations to minimize energetic and physiological stress imposed by the highly reducing, carbon-limited conditions in environments impacted by the geological process of serpentinization. These include enrichment of genes (e.g., CODH, MCR, ACS) that allow for use of substrates made available by the process of serpentinization (e.g., CO, HCOO^−^) and metabolic characteristics to overcome the limited availability of inorganic carbon in these systems. This potentially includes the assimilation of a greater fraction of metabolized carbon from C1 compounds among communities inhabiting hyperalkaline waters when compared with alkaline waters, a characteristic that may allow cells to overcome carbon limitation associated with the former while supporting their energy metabolism with other substrates (e.g., H_2_). Moreover, communities exhibited genomic characteristics that may function to minimize energetic stress imposed by highly reducing, carbon-limited conditions in hyperalkaline waters. This includes a decrease in the genome size of populations (genome streamlining) inhabiting hyperalkaline waters when compared with alkaline waters, as well as a decrease in the Z_c_ in inferred proteomes, a characteristic that may reduce energetic costs of protein synthesis.

## Supplementary information

Supplementary Material

Supplementary Table 5
