# Supplementary material for: Physiological adaptations to serpentinization in the Samail Ophiolite, Oman
Source: ISME J. 2019 Mar 12;13(7):1750–62. doi: 10.1038/s41396-019-0391-2 (PMC6588467; doi:10.1038/s41396-019-0391-2)
Supplement: Supplementary file 1 — Supplementary Material [file 41396_2019_391_MOESM1_ESM.docx]

**SUPPLEMENTAL ONLINE MATERIALS**

**SUPPLEMENTAL FIGURES**

**
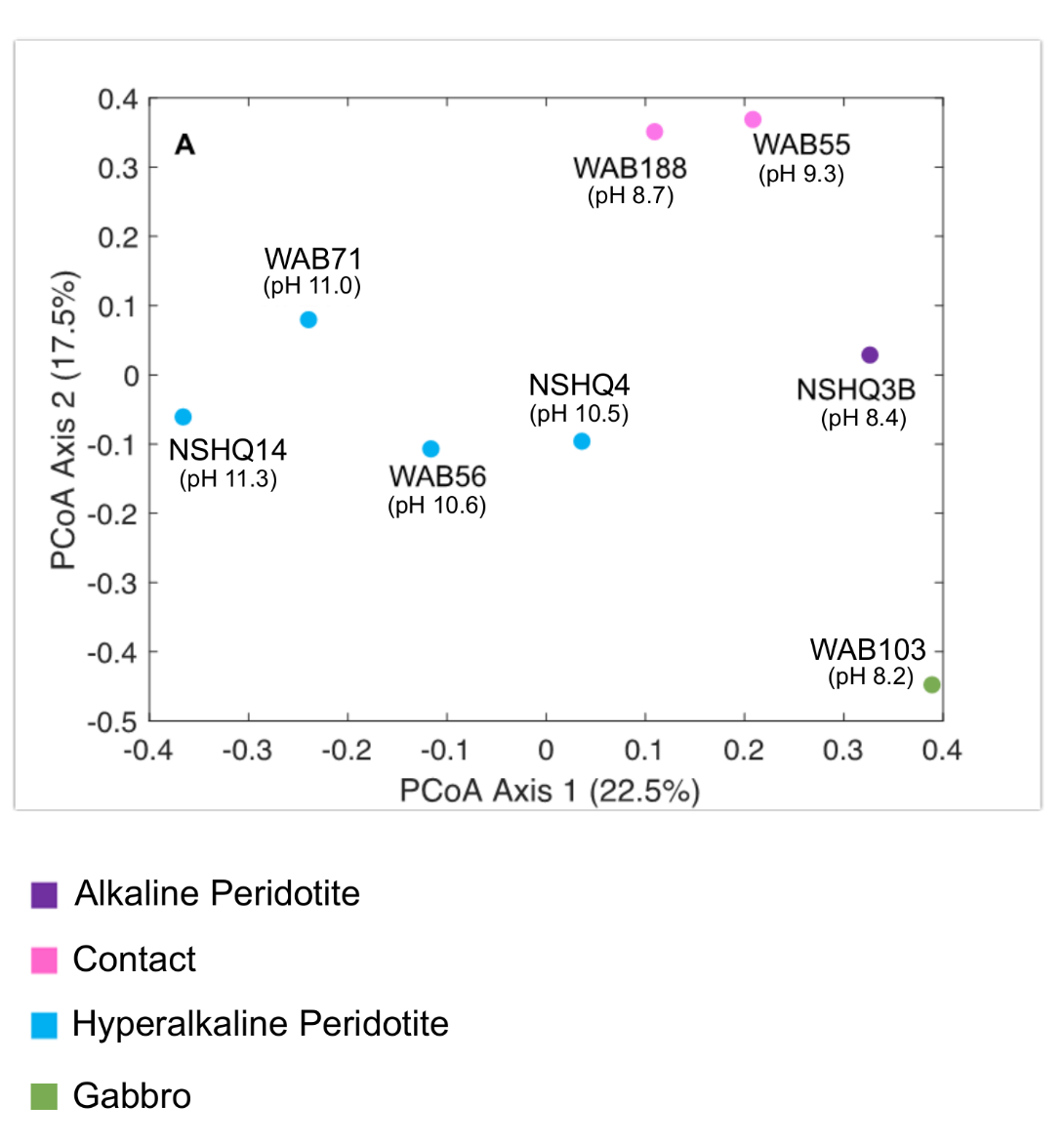
**

**Fig. S1.** Eight metagenome assemblies were produced from eight subsurface wells using the same DNA fraction as was used for 16S rRNA gene sequencing in *Rempfert et al.,* *2017* (1). The pH of the water hosting each community at the time of sampling is listed below the well name, as reported in *Rempfert et al.,* 2017 (1). Metagenome assembly and analysis of 2015 metagenomes is the same as for 2017 metagenomes, except 2015 metagenomes were quality filtered and trimmed of adapters using Trimmomatic v.0.36 and the following parameter settings: LEADING:3 TRAILING:3 SLIDINGWINDOW:4:15 MINLEN:50 (2). Ordination of the 2015 metagenomes is based on dissimilarity in all protein-coding gene homolog families rather than just those annotated as being involved in ‘Metabolism.’ Comparison of ordinations with all protein coding gene homolog families of the 2017 metagenomes and those annotated as being involved in ‘Metabolism’ yielded nearly identical results (data not shown).

**SUPPLEMENTAL TABLES.**

**Table S1.** **Metagenome assembly statistics for 2015 samples.** Eight metagenome assemblies were produced from DNA extracted from filtered biomass collected from eight subsurface wells in 2015.

|  | WAB103 | NSHQ3B | WAB188 | WAB55 | NSHQ4 | WAB56 | WAB71 | NSHQ14  (50m) |
| --- | --- | --- | --- | --- | --- | --- | --- | --- |
| Size (Mbp) | 6.71 | 3.36 | 14.67 | 12.48 | 5.46 | 6.47 | 23.56 | 11.32 |
| Contigs >500 bp | 8,445 | 3,875 | 8,349 | 13,014 | 6,251 | 5,261 | 15,964 | 3,693 |
| Contigs  >10 kbp | 1 | 0 | 184 | 0 | 0 | 0 | 11 | 208 |
| Largest contig (bp) | 23,649 | 6,070 | 83,349 | 10,388 | 7,755 | 18,065 | 64,983 | 144,968 |
| N50 (bp) | 742 | 850 | 2,724 | 978 | 855 | 1,534 | 2,052 | 6,788 |
| Protein-coding genes | 6,151 | 3,539 | 14,949 | 14,219 | 5,436 | 6,694 | 23,519 | 11,216 |

**Table S2. Metagenome assembly statistics for 2017 samples.** Eight metagenome assemblies were produced from DNA extracted from filtered biomass collected from seven subsurface wells in 2017.

|  | WAB104 | WAB105 | WAB188 | WAB55 | NSHQ04 | WAB71 | NSHQ14B  (50m) | NSHQ14C  (85m) |
| --- | --- | --- | --- | --- | --- | --- | --- | --- |
| Size (Mbp) | 128.95 | 60.57 | 233.69 | 137.23 | 151.80 | 87.17 | 129.50 | 72.70 |
| Contigs >500 bp | 54,250 | 23,170 | 67,947 | 51,969 | 54,133 | 36,106 | 40,614 | 23,055 |
| Contigs  >10 kbp | 989 | 434 | 3838 | 1352 | 1651 | 633 | 1711 | 986 |
| Largest contig (bp) | 351,116 | 364,202 | 341,956 | 122,697 | 167,295 | 107,801 | 205,427 | 148,789 |
| N50 (bp) | 2,534 | 2,926 | 5,567 | 3,118 | 3,421 | 2,694 | 4,249 | 4,262 |
| Protein-coding genes | 136,597 | 63,112 | 235,265 | 143,326 | 151,456 | 90,312 | 131,167 | 73,525 |

**Table S3. Planktonic cell densities (cells mL^-1^) in subsurface well water samples from the Samail Ophiolite.** The average (Avg) and standard deviations (SD) associated with reported cell densities were calculated for three replicate subsamples that were each enumerated in a minimum of 10 fields of view.

|  | WAB105 | WAB104 | WAB55 | WAB71 | NSHQ14C  (85m) |
| --- | --- | --- | --- | --- | --- |
| Cells mL^-1^  (Avg) | 3.77 x 10^5^ | 4.03 x 10^5^ | 7.28 x 10^5^ | 2.58 x 10^5^ | 1.16 x 10^5^ |
| Cells mL^-1^  (SD) | 1.51 x 10^5^ | 1.21 x 10^5^ | 1.38 x 10^5^ | 9.07 x 10^4^ | 4.37 x 10^4^ |

**Table S4. Maximum potential rates of biological assimilation and dissimilation (oxidation/reduction) of select one carbon compounds by planktonic microbial communities in well water samples collected from the Samail Ophiolite.** Maximum potential rates of biological substrate transformation were determined via microcosm assays using well waters collected from the Samail Ophiolite, with rates of abiological assays subtracted from biological assays. The average maximum rates of substrate transformation observed in triplicate abiological controls were subtracted from triplicate biological assays (Avg), and their combined standard deviations (SD) are presented. *P*-values were determined between biological assays and abiological controls via Student’s t-test assuming unequal variance for each condition.

| WAB105 WAB104 WAB55 WAB71 NSHQ14C  (85m) | | | | | | |
| --- | --- | --- | --- | --- | --- | --- |
| Formate (HCOO^-^) Oxidation to carbon dioxide (CO_2_) | | | | | | |
| Avg Maximum Rate  (pmol mL^-1^ day^-1^) | | 13138 | 5171 | 1321 | 810 | 309 |
| SD | 2143 | | 3173 | 580 | 221 | 129 |
| *P*-value | 0.01 | | 0.21 | 0.04 | 0.03 | 0.07 |
| HCOO^-^ Assimilation to Biomass | | | | | | |
| Avg Max Rate  (pmol mL^-1^ day^-1^) | 143 | | 79 | 148 | 220 | 230 |
| SD | 43 | | 18 | 66 | 22 | 97 |
| *P*-value | 0.02 | | 0.01 | 0.07 | <0.01 | 0.08 |
| Carbon Monoxide (CO) Oxidation to CO_2_ | | | | | | |
| Avg Max Rate  (pmol mL^-1^ day^-1^) | 148 | | 179 | 134 | 57 | 62 |
| SD | 43 | | 72 | 48 | 23 | 12 |
| *P*-value | 0.02 | | 0.07 | 0.05 | 0.07 | <0.01 |
| CO Assimilation to Biomass | | | | | | |
| Avg. Max Rate  (pmol mL^-1^ day^-1^) | 4 | | 5 | 57 | 6 | 2 |
| SD | 1 | | 2 | 8 | 1 | 2 |
| *P*-value | 0.04 | | 0.04 | <0.01 | <0.01 | 0.09 |
| Bicarbonate (HCO_3_^-^) Reduction to Methane (CH_4_) | | | | | | |
| Avg Max Rate  (pmol mL^-1^ day^-1^) | 1057 | | 2267 | 157 | 26 | 18 |
| SD | 273 | | 543 | 91 | 15 | 6 |
| *P*-value | 0.03 | | 0.03 | 0.13 | 0.13 | 0.04 |
| HCO_3_^-^ Assimilation to Biomass | | | | | | |
| Avg Max Rate  (pmol mL^-1^ day^-1^) | 28 | | 69 | 29 | 56 | 70 |
| SD | 8 | | 32 | 18 | 19 | 62 |
| *P*-value | 0.04 | | 0.09 | 0.12 | 0.01 | 0.21 |

**SUPPLEMENTAL METHODS.**

**Site Description and Water Sampling.** Well NSHQ4 was previously classified as both a “contact” and a “hyperalkaline peridotite” type well, due primarily to its proximity to the crust-mantle boundary and the high pH of its waters, respectively (1). However, NSHQ4 is considered here to be a hyperalkaline peridotite well because it displays geochemical characteristics consistent with other hyperalkaline well waters (Tables 2 and 3).

Samples for measurement of dissolved inorganic carbon (DIC) concentrations were injected through 0.22 µm polycarbonate syringe filters into butyl-stoppered vials that had previously been evacuated, acid-washed, and ashed. Two liters of water from each well were also collected in chemically inert, non-permeable five-liter Cali-5-bond sampling bags equipped with a stopcock for use in cell enumeration and in substrate transformation assays (described below). Prior to collection of waters, the bag was flushed with water, water was dispensed, and the bag was evacuated using a syringe. This was repeated a total of three times for each water sample.

**Geochemical Analyses.** DIC concentrations were quantified by transferring aliquots of water from butyl-stoppered glass sampling vials to helium-purged Exetainer^®^ tubes (Labco, Ceredigion, UK). Total DIC was converted to CO_2_ for analysis by addition of boiled 85% phosphoric acid (H_3_PO_4_) and were equilibrated prior to analysis using a Delta V Isotope Ratio Mass Spectrometer equipped with a Thermo Gasbench II gas preparation and introduction system (Thermo Fisher Scientific). H_2_ and CH_4_ were separated using a 2 m by 1 mm ID micropacked ShinCarbon ST column (Restek Corporation, Bellefonte, PA) and concentrations were determined using an SRI 8610C Gas Chromatograph (SRI Instruments, Torrance, CA). Gases were measured concurrently with a thermal conductivity detector and a flame ionization detector. IC analyses of major anions were conducted on a Dionex ICS-90 (Dionex Corp., Sunnyvale, CA). Correlational analyses between pH and biological parameters were performed via Pearson linear regressions.

**DNA Extraction and Shotgun Metagenomic Sequencing.** DNA from each sample was subjected to ‘tagmentation’ reactions and limited-cycle amplification prior to bead-cleaning and normalization. Libraries were checked for amplification and size distribution on an Agilent 2100 Bioanalyzer with the Agilent DNA 7500 assay (Santa Clara, CA). Products were pooled at equimolar concentrations and sequenced on the Illumina HiSeq 2500 Rapid Run platform (2x250 bp). DNA was sequenced on two separate lanes on the HiSeq platform, and the duplicate fastq paired-end files were concatenated prior to further analysis. Raw sequence reads were quality filtered and trimmed of adapters using PEAT (3). Filtered reads were then assembled using MEGAHIT v.1.1.1 (4) using a range of k-mers between k=21 and k=141. MetaQUAST v.4.3 (5) was used to assess the quality of the assemblies with differing k-mer values and the assembly with the best assembly statistics was used in further analyses. Reads from each metagenome were then mapped to the assembled contigs using Bowtie2 v.2.2.9 (6) to obtain sequencing depth coverage profiles. Proteins from the assembled contigs were identified with Prodigal v.2.6.3 (7) and annotated using Prokka v.1.1.1 (8) with specification of the --metagenome parameter. Contigs were binned into draft metagenome assembled genomes (MAGs) based on tetranucleotide frequencies and coverage profiles using the MetaBAT software package (v. 0.26.3) (9).

**Microcosm Assays.** ^14^C-labeled CO was prepared via dehydration of ^14^C-labeled sodium formate (Na^14^COOH) using previously described methods (10). We assumed quantitative conversion of ^14^HCOO^-^ to ^14^C-CO and added 5 µCi to unlabeled CO to achieve a final concentration of 1 mM total CO in each serum vial.

Microcosm vials were sacrificed for analysis following two, four, six, and eight weeks of incubation at 37°C. Abiological controls were incubated for eight weeks prior to sacrifice and analysis. Disintegrations per minute for all assays were converted to rates of uptake as previously described (11). Rates of CO_2_ and CH_4_ production were normalized to total headspace volume as well as the ratio of radiolabeled to non-radiolabeled substrates. Rates observed in abiological controls were subtracted from those of biological assays to arrive at the rates of substrate transformation attributable to biology. Maximum biological rates, reported as mols mL^-1^ day^-1^, were calculated from data collected over the course of the incubation period. Rates of substrate transformation in biological assays were compared at each timepoint for each substrate to abiological controls using two-tailed Student’s t-tests assuming unequal variance. Maximum potential rates of substrate utilization attributable to microorganisms in each well were then compared with pH and *in situ* cell densities using Pearson linear regressions.

**REFERENCES**

1. Rempfert KR, Miller HM, Bompard N, Nothaft D, Matter JM, Kelemen P *et al*. Geological and geochemical controls on subsurface microbial life in the Samail Ophiolite, Oman. *Front Microbiol* 2017; 8.

2. Bolger AM, Lohse M, Usadel B. Trimmomatic: a flexible trimmer for Illumina sequence data. *Bioinformatics* 2014; 30: 2114-2120.

3. Li YL, Weng JC, Hsiao CC, Chou MT, Tseng CW, Hung JH. PEAT: an intelligent and efficient paired-end sequencing adapter trimming algorithm. *BMC Bioinformatics* 2015; 16: S2.

4. Li D, Liu CM, Luo R, Sadakane K, Lam TW. MEGAHIT: an ultra-fast single-node solution for large and complex metagenomics assembly via succinct de Bruijn graph. *Bioinformatics* 2015; 31: 1674-1676.

5. Mikheenko A, Saveliev V, Gurevich A. MetaQUAST: evaluation of metagenome assemblies. *Bioinformatics* 2016; 32: 1088-1090.

6. Langmead B, Salzberg SL. Fast gapped-read alignment with Bowtie 2. *Nat Methods* 2012; 9: 357-359.

7. Hyatt D, Chen GL, Locascio PF, Land ML, Larimer FW, Hauser LJ. Prodigal: prokaryotic gene recognition and translation initiation site identification. *BMC Bioinformatics* 2010; 11: 119.

8. Seemann T. Prokka: rapid prokaryotic genome annotation. *Bioinformatics* 2014; 30: 2068-2069.

9. Kang DWD, Froula J, Egan R, Wang Z. MetaBAT, an efficient tool for accurately reconstructing single genomes from complex microbial communities. *PeerJ* 2015; 3: e1165.

10. Bonam D, Murrell SA, Ludden PW. Carbon monoxide dehydrogenase from Rhodospirillum rubrum. *J Bacteriol* 1984; 159: 693-699.

11. Urschel MR, Kubo MD, Hoehler TM, Peters JW, Boyd ES. Carbon source preference in chemosynthetic hot spring communities. *Appl Environ Microbiol* 2015; 81: 3834-3847.
